# Hesitancy towards R21/Matrix-M malaria vaccine among Ghanaian parents and attitudes towards immunizing non-eligible children: a cross-sectional survey

**DOI:** 10.1186/s12936-024-04921-2

**Published:** 2024-05-12

**Authors:** Mohamed Fakhry Hussein, Frank Kyei-Arthur, Marina Saleeb, Sylvester Kyei-Gyamfi, Theophilus Abutima, Ignatius Great Sakada, Ramy Mohamed Ghazy

**Affiliations:** 1 Occupational Health and Industrial Medicine Department, High Institute of Public Health, Alexandria University, Alexandria, 21561 Egypt; 2 Department of Environment and Public Health, the University of Environment and Sustainable Development, Somanya, Ghana; 3 Biostatistics Department, MARS-GLOBAL, London, WC2H 9JQ UK; 4 Department of Children, Ministry of Gender, Children and Social Protection, Accra, Ghana; 5 Department of Sociology and Anthropology, University for Development Studies, Nyankpala Campus, Nyankpal, Ghana; 6 Department of Population, Family and Reproductive Health, Kwame Nkrumah University of Science and Technology, Kumasi, Ghana; 7 Department of Family and Community Medicine, College of Medicine, King Khalid University, Abha, Saudi Arabia; 8 Tropical Health Department, High Institute of Public Health, Alexandria University, Alexandria, 21561 Egypt

**Keywords:** Malaria, Vaccine hesitancy, Parent Attitudes about Childhood Vaccination Scale, R21/Matrix-M vaccine, Ghana, Health policy planning

## Abstract

**Background:**

The newly developed malaria vaccine called “R21/Matrix-M malaria vaccine” showed a high safety and efficacy level, and Ghana is the first country to approve this new vaccine. The present study aimed to evaluate the rate of vaccine hesitancy (VH) towards the newly developed malaria vaccine among parents who currently have children who are not eligible for the vaccine but may be eligible in the near future. Additionally, the study aimed to identify the factors that could potentially influence VH.

**Methods:**

A cross-sectional survey using both online-based questionnaires and face-to-face interviews was conducted in Ghana from June to August 2023. The survey specifically targeted parents of ineligible children for vaccination, including those aged less than 5 months or between 3 and 12 years. The Parent Attitudes about Childhood Vaccination (PACV) scale was used to assess parental VH.

**Results:**

A total of 765 people participated in this study. Their median age was 36.0 years with an interquartile range of 31.0–41.0 years, 67.7% were females, 41.8% completed their tertiary education, 63.3% were married, 81.6% worked in non-healthcare sectors, and 59.7% reported that their monthly income was insufficient. About one-third (34.5%) of the parents were hesitant to give their children the R21/Matrix-M malaria vaccine. The following predictors were associated with VH: working in the healthcare sector (adjusted odds ratio (AOR) = 0.50; 95% confidence interval (CI) 0.30–0.80; p = 0.005), having the other parent working in the healthcare sector (AOR = 0.54; 95% CI 0.30–0.94; p = 0.034), and not taking scheduled routine vaccinations (AOR = 1.90; 95% CI 1.27–2.84; p = 0.002).

**Conclusions:**

Addressing VH is crucial for optimizing R21/Matrix-M vaccine coverage in Ghana's malaria control strategy. By tackling VH issues, Ghana can effectively safeguard children's health in malaria-prone areas.

**Supplementary Information:**

The online version contains supplementary material available at 10.1186/s12936-024-04921-2.

## Background

Malaria is a severe febrile disease caused by *Plasmodium* parasites. It can be life-threatening if not promptly diagnosed and treated. The female *Anopheles* mosquito serves as the vector for transmitting the disease [1]. The worldwide estimate of malaria deaths was 625,000 deaths in 2020, compared to 568,000 deaths in 2019, before the coronavirus disease 2019 (COVID-19) pandemic. Of these deaths, about 67% were children under five years of age. The rise in malaria incidence and deaths can be attributed to the disruption in malaria treatment and control services during the COVID-19 pandemic [1–3].

Sub-Saharan Africa is one of the most affected areas by malaria. More than 90% of global deaths due to malaria infections occur in this region [3]. One of the recent efficient tools to prevent malaria is vaccination. The RTS, S/AS01 vaccine, usually known as Mosquirix™, is the first approved malaria vaccine that reduces malaria infection in young children [3]. In October 2021, the World Health Organization (WHO) approved and recommended the use of this vaccine for children living in areas with moderate to high malaria transmission. Endemic countries started to roll out the malaria vaccine through routine vaccination services. Through this campaign, more than 900,000 children had received 1 dose or more of the Mosquirix™ vaccine by the end of January 2022 [3–5]. The Mosquirix ™ vaccine had an effectiveness of 25.9% (95% CI 19.9 to 31.5) among children between the ages of 6 and 12 weeks and 36.3% (95% CI 31.8 to 40.5) among children between the ages of 5 and 17 months against malaria [6].

A new malaria vaccine, called the "R21/Matrix-M malaria vaccine", has demonstrated high levels of safety and efficacy. Following a large phase III trial, it has been found that the new vaccine is more effective than its predecessor, with an efficacy rate of 70–80%. In 2023, Ghana made history by becoming the first country to approve the R21/Matrix-M malaria vaccine, as authorized by the Ghana Food and Drugs Authority. The vaccine is intended for children aged 5 to 36 months -the group at highest risk of malaria infection and mortality [7].

The national malaria vaccination programme in Ghana started in May 2019 across seven regions. By December 2022, 1.4 million doses of the vaccine had been given to children, with 459,446 children getting at least one dose and 184,418 children completing all four doses. The government of Ghana planned to extend the national malaria vaccine campaign to vaccinate more children [8].

These malaria vaccines were projected to protect tens of thousands of people yearly [7]. However, one of the main obstacles to the succession of the vaccination program is vaccination hesitancy (VH), which is “*the reluctance or refusal to vaccinate despite the availability of vaccines”* [9]*.* This could be due to a lack of confidence in the safety and efficacy of the new vaccine, misinformation, or inefficient delivery of vaccination services, which subsequently leads to inefficient malaria control [5, 10, 11].

Improving vaccination systems necessitates a thorough understanding of the root causes of VH. Utilizing validated tools enables us to identify and address the underlying reasons for  VH effectively, thereby improving vaccine coverage [12]. The Parent Attitudes about Childhood Vaccines (PACV) scale has demonstrated both internal and external validity in evaluating parental VH towards vaccinating their children [13, 14].

The hypothesis for this study is that parental attitudes towards R21/Matrix-M malaria vaccine will influence their willingness to vaccinate their children, particularly those currently deemed non-eligible. The urgent need to know the causes of VH towards the newly introduced vaccine is crucial to know the national attitude towards it, hence enacting a specific, well-designed program targeting hesitant people to decrease the refusal rate for the R21/Matrix-M malaria vaccine. Assessing the attitude of parents towards the R21/Matrix-M malaria vaccine is important, especially considering the possibility of extending its recommendation to children who are currently considered non-eligible. Understanding parental attitudes will help us to be well prepared for potential changes in eligibility criteria. Additionally, it serves as an indirect indicator of parents' overall stance on vaccination, including children who are eligible.

## Methods

### The aim

The aim of this study was to assess hesitancy towards the new R21/Matrix-M malaria vaccine among parents of children who could potentially be eligible for vaccination. Additionally, the study aimed to identify factors influencing this attitude.

### Study setting and design

A cross-sectional survey using both online-based questionnaires and face-to-face interviews was conducted in Ghana from June to August 2023.

### Sample size, sampling technique, and target population

The minimum required sample size for assessing hesitancy  towards the R21/Matrix-M malaria vaccine was calculated using EPI-INFO software, considering a VH rate of 50%, a margin of error of 5%, a confidence interval of 95%, and a study power of 80%. The initial calculation yielded a minimum sample size of 384. To account for potential non-responses, an additional 10% was added, resulting in a final sample size of 423 participants. Convenience and snowball sampling techniques were used to recruit the required sample size. Participants were included if they were parents or carer givers of one or more children aged either less than 5 months or between 3 and 12 years (non-eligible children for the R21/Matrix-M malaria vaccination as defined by Ghana government).

### Data collection method and tools

A pre-designed pre-coded structured questionnaire was to collect the data (Additional file 1). The questionnaire was divided into three parts: the first part of the questionnaire collected socio-demographic data including age, sex, nationality, residence, marital status, occupation, education, income, number, and gender of his/her children. The second part of the study collected data about health-related conditions, with a particular focus on preventive measures against malaria. Participants were asked about various methods they used to mitigate the risk of malaria transmission, such as using skin repellents, screening their houses, employing pesticides, utilizing chemoprophylaxis, sleeping under mosquito nets, and wearing long protective clothing. In addition to preventive measures, participants were asked about their personal experiences with malaria. They were asked whether any of their relatives had died from malaria and whether they themselves had ever contracted the disease. Moreover, participants were requested to provide details regarding their family composition. Specifically, they were asked to specify the number of children they have and to disclose the age and sex of the youngest non-eligible child for the R21/Matrix-M malaria vaccine (the youngest child was selected, as the younger the child, the more severe the malaria infection, so the child would have more benefits from the vaccine, and could be eligible in the near future).

Information regarding the age and profession of the other parents of the child under investigation was collected. Data about antenatal care for the youngest child and if the child had a chronic disease was included. The child's previous malaria infection as well as the source of information about vaccination were asked. Participants were asked if their youngest child had received compulsory vaccinations. Data about whether their children had received the Mosquirix™ vaccine was gathered as well. Lastly, the study inquired about whether the individual would give their children the Mosquirix or R21/Matrix-M vaccine, or rather prefer not to administer any malaria vaccine at all. The third part assessed the parental VH to give the R21/Matrix-M vaccine to their children through PACV scale. This validated scale consisted of 15 questions. Each question has a score: hesitant response takes 2 points, ‘don’t know or not sure’ takes 1 point, and non-hesitant response takes 0 point. The participant was considered hesitant if his/her score was fifty or above, and non-hesitant if his/her score was less than fifty (have positive attitude towards the new vaccine) [13]. The PACV had been extensively used to assess parental VH towards different vaccines including COVID-19 and seasonal influenza [15–18].

Before data collection, a pilot study was conducted to evaluate the feasibility and accessibility of the survey. Each study participant was requested to invite at least five participants. The purpose was to assess the time required to complete the questionnaire and the clarity of the questions. Through this process, researchers were able to calculate the average time required to complete the survey and determine the response rate. Moreover, the pilot study helped us to refine the survey instrument and ensure its effectiveness. Ninety-seven responses out of 130 questionnaires sent were received (the overall response rate was 75%). The pilot study data was excluded from the main research analysis. The time taken to fill the questionnaire was 5 to 14 min. Then,  the questionnaire was distributed through various channels, including email, WhatsApp, and multiple social media platforms such as Facebook and Twitter. Additionally, face-to-face interviews were conducted using a similar questionnaire format.

### Ethical considerations

The researchers got approval from the Ethical Committee of the High Institute of Public Health, Alexandria University, Egypt (IRB number: 00013692), prior to starting the study. The objectives and potential benefits of the research were clearly outlined at the beginning of the questionnaire. Written consent was obtained from all study participants, who were assured of their right to withdraw from the survey at any point before completion. The researcher adhered to the International Guidelines for Research Ethics as delineated in the Declaration of Helsinki and its subsequent revisions. Participants were assured that all information provided would be used solely for the purposes of the study. Furthermore, respondents were guaranteed confidentiality and anonymity regarding their responses.

### Statistical analysis

For normally distributed data, quantitative data were presented as mean and standard deviation (SD). Conversely, skewed data were presented as median and interquartile range (IQR). Categorical data were presented as numbers and percentages. To explore the association between categorical variables and the attitude towards R21/Matrix-M vaccine (hesitant vs. non-hesitant), Pearson’s chi-square test or Fischer’s exact test were used. Fischer’s exact test was used only if the assumption of Pearson chi-square test was violated (when more than 20% of the cells had expected frequencies less than 5). The Mann–Whitney test was used to compare continuous data across PACV status since all the numerical variables were not normally distributed. Both univariate and multivariate logistic regression models were built to find the independent variables influencing VH among parents. The backward selection method of the variables was used, in which variables with the highest p-values were removed from the full model one by one until a model with the highest number of significant p-values was reached. Furthermore, assumptions tested for the multivariate model included the Hosmer Lemeshow test to measure the goodness of fit as well as the c-statistic to show the predictive ability of the model. The multicollinearity between the variables was tested by ensuring that the variance inflation factor (VIF) was lower than the value of 10. If applicable, an inferential analysis was deemed statistically significant if the p-value was less than 0.05.

## Results

### Socio-demographic characteristics of parents and children

The current study included 765 participants with a median (IQR) age of 36.0 (31. 41) years, 67.7% were females, 96.4% were Ghanaians, 67.5% lived in urban areas, 71.6% lived in coastal zone, 41.8% completed their tertiary education, 63.3% were married, 81.6% worked in non-healthcare sectors, and 59.7% reported insufficient monthly income, and 12.9% had lost relatives due to malaria infections. Most of the respondents had malaria infection before (86.8%). Regarding the data about children, 44.4% of the parents reported that they had children aged from 3 years to less than 6 years old, 51.8% of the children were males, 94.3% of the mothers attended antenatal care for their youngest child, 12.7% reported their youngest child suffered from chronic diseases, and 66.1% of the children had malaria infection before (Table 1).

**Table 1:** Demographic characteristics of the participants

Socio-demographic characters of the study participants	N = 765 n (%)
Sex	
Female	518 (67.7%)
Male	247 (32.3%)
Age in years	
Median (IQR)	36.0 (31.0.- 41.0)
Nationality	
Ghanaian	738 (96.4%)
Other nationality	27 (3.6%)
Now I am living	
In Ghana	751 (98.2%)
Outside Ghana	14 (1.8%)
Place of residence	
Rural area	249 (32.5%)
Urban area	516 (67.5%)
Located in which ecological zone	
Coastal zone (Western North, Western, Central, Greater Accra, Volta and Oti regions)	548 (71.6%)
Forest zone (Bono, Bono East, Ahafo, Ashanti, and Eastern regions)	118 (15.4%)
Savannah zone (Northern, Upper East, Upper West, Savannah, Northeast regions)	99 (13.0%)
Level of education completed	
Illiterate	55 (7.2%)
Primary education	40 (5.2%)
Middle education	270 (35.3%)
Technical education	80 (10.5%)
Tertiary education	320 (41.8%)
Marital status	
Married	484 (63.3%)
Single	169 (22.1%)
Divorced	88 (11.5%)
Widow	24 (3.1%)
Occupation	
Healthcare sector	141 (18.4%)
Non-healthcare sector	624 (81.6%)
Income	
Not enough	457 (59.7%)
Enough	308 (40.3%)
Had malaria infection before	
Yes	664 (86.8%)
No	77 (10.1%)
I do not know	24 (3.1%)
Had relatives died from malaria	
Yes	99 (12.9%)
No	477 (62.4%)
I do not know	189 (24.7%)
Number of children < 18 years	
Median (IQR)	2 (1, 3)
Age of your youngest child	
Less than 5 months	154 (20.1%)
3–6 years old	340 (44.4%)
6–12 years	271 (35.5%)
Gender of your youngest child	
Female	369 (48.2%)
Male	396 (51.8%)
Age of the other parent of your youngest child in years	
Median (IQR)	38 (32, 43)
Occupation of the other parent	
Healthcare sector	93 (12.2%)
Non-Healthcare sector	672 (87.8%)
Attended antenatal care for your youngest child	
Yes	721 (94.3%)
No	30 (3.9%)
I do not know	14 (1.8%)
Your youngest child is suffering from chronic disease	
Yes	97 (12.7%)
No	668 (87.3%)
Your youngest child had malaria infection before	
Yes	506 (66.1%)
No	230 (30.1%)
I do not know	29 (3.8%)

### Vaccination status of the children under investigation

Concerning vaccination status, about three-quarters (74.0%) of the parents stated that their children took the scheduled vaccines up to their ages, 38.7% of them confirmed their children received previous malaria vaccine, and 44.2% reported they would give the R21/Matrix-M malaria vaccine to their children, while 47.1% said they preferred the Mosquirix™ (Table 2).

**Table 2:** Vaccination status of the children under investigation

Vaccination status of the children	N = 765 n (%)
Your youngest child has received scheduled vaccine	
Yes	566 (74.0%)
No	199 (26.0%)
Your children received previous malaria vaccine	
Yes	296 (38.7%)
No	390 (51.0%)
I do not know	79 (10.3%)
The type of malaria vaccine you prefer to give to your child	
The new R21/Matrix-M Malaria vaccine	338 (44.2%)
The old RTS, S/AS01 (Mosquirix)	360 (47.1%)
I do not know/I will not vaccinate my child	67 (8.7%)

### Parental hesitancy to the R21/Matrix-M malaria vaccine regarding different socio-demographic characteristics

Table 3 shows a comparison of the socio-demographic data between the two groups which were hesitant parents (n = 264, 34.5%) and non-hesitant parents (n = 501, 65.5%). Sex was found to be significantly associated with parental VH, with 62.5% of hesitant participants being females compared to 37.5% males (p = 0.025). Similarly, living zone and occupation influenced parental VH; 74.6% of non-hesitant parents lived in coastal areas, whereas about one-third of hesitant parents resided in other ecological zones (p = 0.009). Additionally, 12.5% of vaccine-hesitant parents worked in the healthcare sector compared to 87.5% in non-healthcare sectors (p = 0.002). Moreover, 65.5% of the hesitant group reported insufficient income, while 34.5% deemed it sufficient (p = 0.018). Non-hesitant parents were more likely to report that their children had previously contracted malaria compared to hesitant parents (68.9% vs. 61.0%; p = 0.003). Additionally, a greater proportion of children in the non-hesitant parents' group had received scheduled vaccinations (78.8%) compared to the hesitant parents' group (64.8%) (p < 0.001). Moreover, non-hesitant parents showed a preference for the R21/Matrix-M vaccination (52.1%), whereas hesitant parents favored the Mosquirix™ vaccine (57.6%) (p < 0.001).

**Table 3:** Socio-demographic characteristics distributed across hesitant and non-hesitant participants

Demographic characters of the study participants	Attitude towards R21/Matrix-M malaria vaccine based on PACV score		p-value
	Hesitant,	Non-hesitant,	
	N = 264 (34.50%)	N = 501 (65.50%)	
Sex			
Female	165 (62.5%)	353 (70.5%)	0.025*^a^
Male	99 (37.5%)	148 (29.5%)	
Age in years			
Median (IQR)	36 (31, 41)	36 (30, 41)	0.426 U
Nationality			
Ghanaian	254 (96.2%)	484 (96.6%)	0.779^a^
Other nationality	10 (3.8%)	17 (3.4%)	
Now I am living			
In Ghana	261 (98.9%)	490 (97.8%)	0.401 FE
Outside Ghana	3 (1.1%)	11 (2.2%)	
Place of residence			
Rural area	80 (30.0%)	169 (33.7%)	0.336^a^
Urban area	184 (70.0%)	332 (66.3%)	
Located in which ecological zone			
Coastal zone	174 (65.9%)	374 (74.6%)	0.009*^a^
Forest zone	55 (20.8%)	63 (12.6%)	
Savannah zone	35 (13.3%)	64 (12.8%)	
Level of education completed			
Illiterate	26 (9.8%)	29 (5.8%)	0.057^a^
Primary education	15 (5.7%)	25 (5.0%)	
Middle education	77 (29.2%)	193 (38.5%)	
Technical education	30 (11.4%)	50 (10.0%)	
Tertiary education	116 (43.9%)	204 (40.7%)	
Marital status			
Married	168 (63.6%)	316 (63.1%)	0.526^a^
Single	52 (19.7%)	117 (23.3%)	
Divorced	35 (13.3%)	53 (10.6%)	
Widow	9 (3.4%)	15 (3.0%)	
Occupation			
Healthcare sector	33 (12.5%)	108 (21.6%)	0.002*^a^
Non-healthcare sector	231 (87.5%)	393 (78.4%)	
Income			
Not enough	173 (65.5%)	284 (56.7%)	0.018*^a^
Enough	91 (34.5%)	217 (43.3%)	
Had malaria infection			
Yes	224 (84.8%)	440 (87.8%)	0.240^a^
No	33 (12.5%)	44 (8.8%)	
I do not know	7 (2.7%)	17 (3.4%)	
Had relatives died from malaria			
Yes	35 (13.3%)	64 (12.8%)	0.651^a^
No	159 (60.2%)	318 (63.4%)	
I do not know	70 (26.5%)	119 (23.8%)	
Number of children aged <18 years	2 (1, 3)	2 (1, 3)	0.864 U
Age of your youngest child			
Less than 5 months	59 (22.3%)	95 (19.0%)	0.540^a^
3 to less than 6 years old	114 (43.2%)	226 (45.1%)	
6–12 years	91 (34.5%)	180 (35.9%)	
Gender of your youngest child			
Female	128 (48.5%)	241 (48.1%)	0.920^a^
Male	136 (51.5%)	260 (51.9%)	
Age of the other parent			
Median (IQR)	37 (32, 43)	38 (32, 43)	0.438 U
Occupation of the other parent			
Healthcare sector	22 (8.3%)	71 (14.2%)	0.019*^a^
Non-Healthcare sector	242 (92.7%)	430 (85.8%)	
Attended antenatal care for your youngest child			
Yes	244 (92.4%)	477 (95.2%)	0.273 FE
No	14 (5.3%)	16 (3.2%)	
I do not know	6 (2.3%)	8 (1.6%)	
Your youngest child is suffering from chronic disease			
Yes	35 (13.3%)	62 (12.4%)	0.727^a^
No	229 (86.7%)	439 (87.6%)	
Your youngest child had malaria infection before			
Yes	161 (61.0%)	345 (68.9%)	0.003*^a^
No	85 (32.2%)	145 (28.9%)	
I do not know	18 (6.8%)	11 (2.2%)	
Your youngest child has received scheduled vaccines			
Yes	171 (64.8%)	395 (78.8%)	< 0.001*^a^
No	93 (35.2%)	106 (21.2%)	
Your children received previous malaria vaccine			
Yes	101 (38.3%)	195 (38.9%)	0.350^a^
No	130 (49.2%)	260 (51.9%)	
I do not know	33 (12.5%)	46 (9.2%)	
The type of malaria vaccine given to your child			
The new R21/Matrix-M Malaria vaccine	77 (29.1%)	261 (52.1%)	< 0.001*^a^
The old RTS, S/AS01 (Mosquirix)	152 (57.6%)	208 (41.5%)	
I do not know/I will not vaccinate my child	35 (13.3%)	32 (6.4%)	

U Mann–Whitney test, FE Fischer’s exact test

^a^ Pearson’s Chi-squared test;

^*^Significant p-value < 0.05

### Knowledge and practices of parents towards malaria prevention and sources of information about the vaccine

Approximately two-thirds (67.3%) of the participants reported using insecticides, while 54.5% stated that they lived in homes with screens on all windows and doors. Yet, there was no significant difference between hesitant and non-hesitant people regarding these practices. The majority (81.0%) of the parents depended on healthcare workers to get information about malaria vaccines, 85.6% of non-hesitant parents versus 72.3% of hesitant parents rely on healthcare workers to get this information (p < 0.001). Mass media (41.2%) and friends or neighbors (33.3%) were important sources of information (Table 4).

**Table 4:** knowledge and Practices associated with the new vaccine hesitancy and sources of information about vaccines

Knowledge and Practices related to malaria infection and sources of information about vaccines	(N = 7651)^a^	Attitude towards R21/Matrix-M malaria vaccine based on PACV PACV score		p-value^b^
		Hesitant, N = 264	Non-hesitant, N = 501	
Practices to avoid malaria infection				
I sleep under a mosquito net	388 (50.7%)	124 (47.0%)	264 (52.7%)	0.132
I use insecticides	515 (67.3%)	188 (71.2%)	327 (65.3%)	0.096
I use chemoprophylaxis	100 (13.1%)	29 (11.0%)	71 (14.2%)	0.214
I wear long-sleeved shirts and long pants	210 (27.5%)	74 (28.0%)	136 (27.1%)	0.794
I treat clothes with insect repellent like permethrin	48 (6.3%)	21 (8.0%)	27 (5.4%)	0.164
I live inside home with all windows and doors have screens	417 (54.5%)	140 (53.0%)	277 (55.3%)	0.551
I do not practice any of the above	60 (7.8%)	29 (11.0%)	31 (6.2%)	0.019*
Sources of information about vaccines				
Healthcare professionals	620 (81.0%)	191 (72.3%)	429 (85.6%)	< 0.001*
Mass media	315 (41.2%)	107 (40.5%)	208 (41.5%)	0.792
Social media	227 (29.7%)	83 (31.4%)	144 (28.7%)	0.438
Community leaders	78 (10.2%)	32 (12.1%)	46 (9.2%)	0.201
Friends or neighbors	255 (33.3%)	83 (31.4%)	172 (34.3%)	0.42
Family member	216 (28.2%)	80 (30.3%)	136 (27.1%)	0.356
Scientific books/websites	108 (14.1%)	29 (11.0%)	79 (15.8%)	0.071
I did not hear about it before	55 (7.2%)	30 (11.4%)	25 (5.0%)	0.001*
Reasons why you will give the R21/Matrix-M malaria vaccine to your children				
I trust the local authorities	292 (38.2%)	57 (21.6%)	235 (46.9%)	< 0.001*
It is more effective	157 (20.5%)	34 (12.9%)	123 (24.6%)	< 0.001*
Less cost	195 (25.5%)	33 (12.5%)	162 (32.3%)	< 0.001*
It is safer	192 (25.1%)	44 (16.7%)	148 (29.5%)	< 0.001*
Not applicable/I will not give them the vaccine	138 (18.0%)	71 (26.9%)	67 (13.4%)	< 0.001*
Others	236 (30.8%)	109 (41.3%)	127 (25.3%)	< 0.001*
It is useless/ineffective	29 (3.8%)	17 (6.4%)	12 (2.4%)	0.005*
No sufficient studies were published on its effect	117 (15.3%)	58 (22.0%)	59 (11.8%)	< 0.001*
Reasons why you will not give the R21/Matrix-M malaria vaccine to your children				
Shortage in delivery and vaccination services	49 (6.4%)	18 (6.8%)	31 (6.2%)	0.735
It may have side effects	249 (32.5%)	107 (40.5%)	142 (28.3%)	< 0.001*
Not applicable/I will give them the vaccine	338 (44.2%)	73 (27.7%)	265 (52.9%)	< 0.001*
Others	221 (28.9%)	92 (34.8%)	129 (25.7%)	0.008*

^a^n (%)

^b^Pearson’s Chi-squared test

^*^Significant p < 0.05

### Reasons for parental hesitancy

Concerning reasons for giving the R21/Matrix-M malaria vaccine to the children, only 38.2% of the parents reported they trust the local authorities. Nearly half (46.9%) of non-hesitant participants trust local authorities, compared to 21.6% of hesitant parents with significant difference between the two groups (p < 0.001). However, 32.5% said they would  not give the vaccine to their children because of their fear from its side effects. The higher effectiveness of the R21/Matrix-M malaria vaccine was a significant factor for non-hesitant parents compared to hesitant participants (24.6% vs. 12.9%) (p < 0.001). About one-third (32.3%) of non-hesitant parents considered low cost as a reason for giving the vaccine, compared to 12.5% of hesitant parents (p < 0.001). Non-hesitant participants had higher trust in safety compared to hesitant parents (29.5% vs. 16.7%) (p < 0.001).

A higher percentage of the hesitant group refused to give the new vaccine to their children compared to the non-hesitant group (p < 0.001). Regarding reasons for refusal to give the new vaccine to the children, a higher percentage of hesitant parents reported that the cause was due to insufficient published studies on its effect (22.0%) compared to non-hesitant parents (11.8%) (p < 0.001) as they heard from their friends or healthcare workers. About two-fifths (40.5%) of hesitant parents were afraid of side effects of the new vaccine compared to 28.3% of non-hesitant responders (p < 0.001) (Table 4).

### Adjusted and unadjusted odds ratio of factors related to parental hesitancy towards the R21/Matrix-M malaria vaccine

Living in a forest zone increased the VH compared to living in a coastal zone (crude odds ratio (COR) = 1.88; 95% confidence interval (CI) 1.25–2.81; p = 0.002), but this association became insignificant in the adjusted analysis. Participants who work in the healthcare sector had significantly lower odds of R21/Matrix-M malaria VH by 50% compared to non-healthcare workers (adjusted odds ratio (AOR) = 0.50; 95%CI 0.30–0.80; p = 0.005) in the adjusted analysis. Even the occupation of the other parents of the child could also affect the VH; working of the other parent in the healthcare sector lowered the odds of R21/Matrix-M malaria VH by 46% compared to non-healthcare workers (AOR = 0.54; 95% CI 0.30–0.94; p = 0.034). Having enough income decreased the crude odds of VH by 31% compared to people who had insufficient income (COR = 0.69; 95%CI 0.50–0.94; p = 0.018), but in the adjusted regression model, the association became insignificant. Experience of previous malaria infection for the children elevated the crude odds of VH by 1.41 compared to non-infected children (COR = 1.41; 95% CI 1.04–1.93; p = 0.029).

Moreover, parents whose children did not take the scheduled routine vaccinations had higher odds of R21/Matrix-M malaria VH by 90% compared to those whose children were fully vaccinated till their ages (AOR = 1.90; 95% CI 1.27–2.84; p = 002). Participants who chose to give the new vaccine to their children had lower crude odds of VH by 73% (COR = 0.27; 95%CI 0.16–0.46; p < 0.001) (Table 5).

**Table 5:** Unadjusted and adjusted odds ratio showing the predictors of hesitancy of the parents to vaccinate their children with the R21/Matrix-M malaria vaccine

Dependent: Hesitant parents whose PACV scores are equal to or more than 50	Crude odds ratio	Adjusted odds ratio (95 CI LL–UL) p-value
	(95 CI LL–UL) p-value	
Sex		
Female	– reference	– reference
Male	1.43 (1.04–1.96, **p = 0.026**)	1.24 (0.80–1.92, p = 0.333)
Age in years	1.01 (0.99–1.03, p = 0.523)	1.01 (0.97–1.04, p = 0.692)
Nationality		
Ghanaian	– reference	– reference
Other nationality	1.12 (0.49–2.44, p = 0.779)	1.27 (0.51–3.02, p = 0.595)
Place of residence		
Rural area	– reference	– reference
Urban area	1.17 (0.85–1.62, p = 0.336)	0.91 (0.62–1.33, p = 0.612)
Located in which ecological zone		
Coastal zone	– reference	– reference
Forest zone	1.88 (1.25–2.81, **p = 0.002**)	1.48 (0.92–2.37, p = 0.103)
Savannah zone	1.18 (0.74–1.83, p = 0.481)	0.81 (0.47–1.37, p = 0.443)
Level of education completed		
Technical education or lower	– reference	– reference
Tertiary education	1.14 (0.84–1.54, p = 0.391)	1.32 (0.89–1.98, p = 0.169)
Marital status		
Not married	– reference	– reference
Married	1.02 (0.75–1.40, p = 0.878)	1.01 (0.69–1.48, p = 0.950)
Occupation		
Non healthcare sector	– reference	– reference
Healthcare sector	0.52 (0.34–0.79, **p = 0.002**)	0.50 (0.30–0.80, **p = 0.005)**
Income		
Not enough	– reference	– reference
Enough	0.69 (0.50–0.94, **p = 0.018**)	0.76 (0.53–1.08, p = 0.131)
Had previous malaria infection		
Yes	– reference	– reference
No	1.29 (0.83–1.97, p = 0.249)	0.95 (0.58–1.54, p = 0.835)
Had relatives died from malaria		
Yes	– reference	– reference
No	0.96 (0.62–1.50, p = 0.850)	0.67 (0.41–1.10, p = 0.109)
Number of children with age less than 18 years	0.98 (0.86–1.11, p = 0.710)	0.96 (0.82–1.11, p = 0.569)
Age of your youngest child		
Less than 5 months	– reference	– reference
3 to less than 6 years old	0.81 (0.55–1.21, p = 0.302)	1.01 (0.59–1.73, p = 0.971)
6–12 years	0.81 (0.54–1.23, p = 0.327)	0.82 (0.44–1.54, p = 0.537)
Sex of your youngest child		
Female	– reference	– reference
Male	0.98 (0.73–1.33, p = 0.920)	0.95 (0.68–1.32, p = 0.745)
Age of the other parent of your youngest child		
–	0.99 (0.97–1.01, p = 0.291)	1.00 (0.97–1.03, p = 0.879)
Occupation of the other parent of your youngest child		
Non-Healthcare sector	– reference	– reference
Healthcare sector	0.55 (0.33–0.90, **p = 0.020**)	0.54 (0.30–0.94**, p = 0.034**)
Attended antenatal care for your youngest child		
Yes	– reference	– reference
No	1.63 (0.87–3.00, p = 0.119)	1.10 (0.54–2.21, p = 0.795)
Your youngest child is suffering from chronic disease		
Yes	– reference	– reference
No	0.92 (0.60–1.45, p = 0.727)	0.69 (0.41–1.16, p = 0.154)
Your youngest child had malaria infection before		
Yes	– reference	– reference
No	1.41 (1.04–1.93, **p = 0.029**)	1.10 (0.72–1.68, p = 0.657)
Your youngest child has received scheduled vaccines		
Yes	– reference	– reference
No	2.03 (1.45–2.82, **p < 0.001**)	1.90 (1.27–2.84, **p = 0.002**)
Your children received previous Malaria vaccine		
Yes	– reference	– reference
No	1.03 (0.76–1.40, p = 0.858)	0.82 (0.57–1.19, p = 0.301)
What type of malaria vaccine you give to your child?		
I do not know/	– reference	– reference
I will not vaccinate my child		
The new R21/Matrix-M Malaria vaccine	0.27 (0.16–0.46, **p < 0.001**)	0.86 (0.43–1.74, p = 0.682)
The old RTS, S/AS01 (Mosquirix)	0.67 (0.39–1.13, p = 0.131)	0.92 (0.51–1.63, p = 0.764)

Assumptions for the multivariate model: Hosmer Lemeshow test (p > 0.05)/c-statistic = 0.79/VIF for all the variables < 10. Bold p-value means it is statistically significant as it is less than 0.05

## Discussion

Vaccination is one of the best methods to prevent and control infectious diseases. Low vaccination coverage could be due to VH, which led to an elevation in the risk of infectious diseases transmission, ending in outbreaks or epidemics [10, 12]. The current study assessed the parental VH rate towards the R21/Matrix-M malaria vaccine in Ghana and investigated the associated factors. The PACV questionnaire was used to classify parents into hesitant and non-hesitant groups. About one-third (34.5%) of the parents were hesitant to give their children the R21/Matrix-M malaria vaccine. In summary, parents who work in the medical field were less likely to be hesitant about the R21/Matrix-M vaccine, but parents whose children did not obtain the scheduled vaccinations were more likely to be hesitant.

Similarly, a qualitative Ghanaian study carried out to  address the awareness of the mothers towards the malaria vaccine reported that the participants had a positive attitude towards the vaccine as it reduces hospital admissions and is cost-effective. Health professionals greatly influenced the adoption of the vaccination. However, recommending the vaccine to other carers may be hampered by concerns about unidentified negative effects [19].

*The hesitancy towards malaria vaccination*: The R21/Matrix-M malaria vaccine is an important move towards the elimination of the disease, but acceptance of this vaccine, especially in low- and middle-income countries, may provide a hurdle that needs to be overcome for any vaccination program to be carried out successfully [20]. The current research stated that 34.5% of parents were hesitant to vaccinate their children with the R21/Matrix-M malaria vaccine, while non-hesitant participants who were ready to give the new vaccines to their children accounted for 65.5%. Many studies were held to examine the acceptance rate of the malaria vaccine in low-income countries where malaria is an endemic disease [21–23]. Acceptance rates of the malaria vaccine varied across different studies: from 32.3% in an Ethiopian study conducted by Asmare [21], to 70% in a study by Amin et al*.* in Bangladesh [22], 84.2% in a study by Mtenga et al*.* in Tanzania [23] and the a high acceptance at 95.3% was reported in a meta-analysis of 11 studies conducted in 5 low and middle income countries by Sulaiman et al*.* [24]. In fact, acceptance of vaccination differs according to occupation, religion, and region. The positive attitudes were due to the sense of need for further malaria prevention approaches and the high hopes for the vaccine. They thought that the vaccine could reduce hospital admissions, deaths, the severity of the infection, and treatment costs [21, 24].

*Socioeconomic factors affecting the VH*. In the current study, VH towards the R21/Matrix-M malaria vaccine was significantly higher in the forest zone than in the coastal zone. Those who had enough income had lower VH compared to people with insufficient income. People living in remote areas like forests had problems with immunization service accessibility, transportation availability, and well-established infrastructure, along with sociodemographic differences that could increase VH [24–27]. Troiano et al*.* [28] in a narrative review, stated that those with lower incomes had higher levels of VH. This may be due to the fact that people with low incomes usually have a low level of education and are less concerned about preventive medicine, so their awareness of vaccination is low, leading to a refusal of immunization.

According to the multiple logistic regression model, participants who worked in the healthcare sector had significantly lower VH towards the R21/Matrix-M malaria vaccine compared to those in non-healthcare professions. Healthcare workers appear to be more likely to adopt vaccines since they frequently have higher education, practical experience, and participation in organizations that support immunization. In addition, many medical professionals respond to pandemics and epidemics firsthand. These could tangentially impact their perspective on immunizing their children [29].

Regarding the present study, participants with children who had completed scheduled vaccines had a higher level of acceptance of the R21/Matrix-M malaria vaccine compared to those with children who had not taken the scheduled routine vaccinations. In the same vein, Asmare [21] in a cross-sectional study in Ethiopia, reported that parents' readiness to accept giving a malaria vaccine to their children was substantially associated with their prior experience receiving childhood vaccinations (AOR = 2.673; 95% CI 1.759–4.101).

*Sources of information about vaccination*: As regards the current study, non-hesitant parents depend on healthcare workers to get information about malaria vaccines significantly more than hesitant participants. Besides, mass media and friends constitute important sources of information. These results attract attention to the importance of spreading health messages about the new vaccine through healthcare workers and common channels of information like mass media and social platforms. The more the government invests in delivering the correct information, the lower the hesitancy rate for the R21/Matrix-M malaria vaccine [20, 30].

*Reasons for VH*: The reasons for giving the R21/Matrix-M malaria vaccine to the children in the present study were trust in the local authorities, the higher effectiveness of the new vaccine, and trust in its safety. Similarly, Yeboah et al*.* [5] reported a statistically significant association between the actual malaria vaccine uptake and belief in the vaccination's efficacy. The most important causes of VH, according to present study, were that there are not sufficient published studies about the R21/Matrix-M malaria vaccine, and it may have undiscovered side effects. These results were supported by many previous studies where safety issues, the effectiveness of the vaccine, and a low level of awareness were among the crucial factors contributing to its high VH [31, 32]. These concerns are common for any new vaccine, like vaccines against COVID-19, so intensifying efforts to abolish misconceptions about these new vaccines is pivotal to reduce VH [28, 33]. Each group of people has their own fears or concerns about immunization, which include neighbours’ attitudes, the cost of the vaccine, worry about side effects, and a lack of trust in the local authority. The variety of parental attitudes about vaccinating their children against malaria indicates that malaria vaccination campaigns should not have one message for all audiences but should be modified to match the variety of parents’ beliefs and attitudes [34].

## Limitations and strengths

Some limitations should be addressed for this study. First, the generalizability of the results may be hampered by the use of non-randomized sample techniques. Second, online and interview surveys that depend on individual’s memory and subjective responses have the risk of introducing recall and social desirability biases, which could affect the study findings. Despite these limitations, this study had several advantages. It is the first study to explore parental VH towards R21/Matrix-M. Second, gathering data from a large geographic area using different modalities (an online questionnaire besides the use of face-to-face interview) enhanced the reach for various groups of the community. Additionally, the inclusion of PACV that has been validated enhances the internal consistency of this study. Finally, the thoroughness of the methodology was displayed by the pilot study that was conducted prior to the main information gathering.

## Conclusions

In conclusion, this study underscores the importance of understanding Ghanaian parents' perspectives on the R21/Matrix-M malaria vaccine for ineligible children. Such insight is crucial as the vaccine shows great promise in reducing the incidence and severity of malaria in children. Nearly one third of participants exhibited VH, which was determined by several sociodemographic characteristics. These included being female, employed in non-healthcare sectors, having insufficient income, having a child with a history of malaria, and having a child who had not received scheduled vaccinations. The reasons for giving the R21/Matrix-M malaria vaccine to the children were trust in the local authorities, being more effective, safer, and less costly. Parents cited two main reasons for not administering the R21/Matrix-M malaria vaccine to their children: concerns about insufficient published studies on its effects and potential side effects. To safeguard the health and well-being of children in malaria-prone regions, it is imperative to address these concerns and optimize the effectiveness of the R21/Matrix-M vaccine. This necessitates ongoing educational campaigns, research initiatives, and collaborative efforts, including the implementation of modern health approaches i.e. one health approach [35].

### Supplementary Information

**Additional file 1: **Study questionnaire.

## Data Availability

The datasets used and/or analysed during the current study are available from the corresponding author on reasonable request.

## Abbreviations

AOR: Adjusted odds ratio
CI: Confidence interval
COR: Crude odds ratio
COVID-19: Coronavirus disease 2019
IQR: Interquartile range
PACV: The Parent Attitudes about Childhood Vaccination Scale
RTS, S/AS01 RTS, S, (Mosquirix TM): The old malaria vaccine
R21/Matrix-M vaccine: The new malaria vaccine
SD: Standard deviation
VH: Vaccine hesitancy
VIF: Variance Inflation Factor
WHO: World Health Organization
