# Supplementary material for: Hesitancy towards R21/Matrix-M malaria vaccine among Ghanaian parents and attitudes towards immunizing non-eligible children: a cross-sectional survey
Source: Malar J. 2024 May 12;23:142. doi: 10.1186/s12936-024-04921-2 (PMC11088762; doi:10.1186/s12936-024-04921-2)
Supplement: Supplementary file 1 — Additional file 1: Study questionnaire. [file 12936_2024_4921_MOESM1_ESM.docx]

**Acceptance of the Newly Developed R21/Matrix-M Malaria Vaccine Among Ghanaian Population towards non-eligible children: A Cross-Sectional Survey**

You are invited to participate in research entitled “Acceptance of the Newly Developed R21/Matrix-M Malaria Vaccine Among Ghanaian Population towards non-eligible children: A Cross-Sectional Survey”. This information is crucial to know the attitude towards the new R21/Matrix-M malaria vaccine after the approval of this vaccine by local Ghanaian authorities. It will assess parents’ intention to vaccinate their children by the new malaria vaccine.

Your participation is voluntary and anonymous. You are free to withdraw at any time. This study was approved by the Ethical Committee. The information provided will be used only for research purposes. Confidentiality will be maintained. The survey should take about 5 to 10 minutes to complete. Thank you for sharing your valuable time with us.

If you have any inquiry, please contact the authors.

1. **I accept to be involved in the research.**
   1. Yes, Continue
   2. Do not want to be in the research.

**Please answer the following socio-demographic questions**

1. **Sex**
2. Male
3. Female
4. **Age (by years)**
5. **Nationality**
   1. Ghanaian
   2. Other nationality
6. **Now I am living**
   1. In Ghana
   2. Outside Ghana
7. **Place of residence**
   1. Urban area
   2. Rural area
8. **In which ecological zone are you located?**
   1. Savannah zone (Northern, Upper East, Upper West, Savannah, North East regions)
   2. Coastal zone ( Western North, Western, Central, Greater Accra, Volta and Oti regions)
   3. Forest zone (Bono, Bono East, Ahafo, Ashanti, and Eastern regions).
9. **Level of education completed.**
   1. I did not complete any level of education.
   2. Pre-School
   3. Primary education
   4. JSS/JHS
   5. Middle education
   6. SSS/SHS
   7. Vocational/Technical
   8. Tertiary education
10. **Marital status**
11. Married
12. Single
13. Widow
14. Divorced
15. Separated
16. **Occupation**
    1. Healthcare sector
    2. Non healthcare sector
17. **Income**
    1. Not enough, took a loan and cannot pay.
    2. Not enough, took a loan but can pay back.
    3. Not enough, did not take a loan.
    4. Enough
    5. Enough and can save money
18. **Have you received Malaria infection before?**
    1. Yes
    2. No
    3. I do not know
19. **Did you have relatives died from Malaria?**
    1. Yes
    2. No
    3. I do not know
20. **Practice to avoid Malaria infection**
    1. I sleep under mosquito net
    2. I use insecticides
    3. I use chemoprophylaxis (drugs that prevent disease before appearing of symptoms)
    4. I wear long-sleeved shirts and long pants
    5. I treat clothes with insect repellent like permethrin
    6. I live inside home with all windows and doors have screens
    7. I do not practice any of the above
21. **Do you have children with age less than 18 years?**
    1. Yes
    2. No
22. **How many children with age less than 18 years do you have?**
23. **Age of your youngest child**
    1. Less than 5 months
    2. 5 -11 months
    3. 1 – less than 3 years
    4. 3 – less than 6 years
    5. 6 - 12 years
24. **Sex of your youngest child**
    1. Male
    2. Female
25. **Age of the other parent of your youngest child (in years)**
26. **Occupation of the other parent of your youngest child**
    1. Health care sector
    2. Non healthcare sector
27. **Did you (or the another parent of your child) attend antenatal care for your youngest child?**
    1. Yes, regularly
    2. Yes, sometimes
    3. No
    4. I do not know
28. **Is your youngest child suffering from chronic disease?**
    1. Yes
    2. No
29. **If your youngest child suffers from chronic disease, mention it**
    1. No
    2. Heart disease
    3. Chest disease (like asthma)
    4. Neurological disease (like convulsions, autism, or cerebral palsy)
    5. Blood disease (like hemolytic anemia)
    6. Metabolic disease (like cystic fibrosis)
    7. Endocrine disease (like Diabetes mellitus or thyroid disease)
    8. Liver disease (like liver fibrosis, fatty liver, or liver failure)
    9. Kidney disease
    10. Hearing impairment
    11. Vision impairment
    12. Loss of limb function
    13. Others
30. **Has your youngest child received Malaria infection before?**
    1. Yes
    2. No
    3. I do not know
31. **Source of information about vaccines**
    1. Healthcare professionals
    2. Mass media
    3. Social media
    4. Community leaders
    5. Friends or neighbors
    6. Family member
    7. Scientific books or scientific websites
    8. I did not hear about it before

1. **Did your youngest child receive scheduled vaccines?**
   1. Yes
   2. No
   3. He / she did not take all the scheduled vaccines for his/ her age
2. **Did your children receive previous Malaria vaccine?**
   1. Yes
   2. No
   3. I do not know
3. **Would you give the new Malaria vaccine to your children?**
   1. Yes
   2. No
   3. I am not sure.
4. **What type of Malaria vaccine would you prefer to give to your children?**
   1. The new R21/Matrix-M malaria vaccine
   2. The old RTS, S/AS01 (Mosquirix)
   3. I will not give any Malaria vaccine to my children
   4. I do not know
5. **I will give the new Malaria vaccine to my children because (you can choose more than one answer)**
   1. It is more safe
   2. It is more effective
   3. Less cost
   4. I trust the local health authority
   5. Not applicable "I would not give the new Malaria vaccine to my children "
   6. Others
6. **I would not give the new Malaria vaccine to my children because (you can choose more than one answer)**
   1. It may have side effects
   2. It is useless/ ineffective
   3. No sufficient studies have been published on its effect
   4. Shortage in delivery of vaccination services
   5. Not applicable "I will give the new Malaria vaccine to my children. "
   6. Others

**Questions about the Parent Attitudes about Childhood Vaccines (PACV)**

Please answer the following questions about the scheduled vaccines for your youngest child

1. **Have you ever delayed having your child get a shot for reasons other than illness or allergy?**
   1. Yes
   2. No
   3. I do not know
2. **Have you ever decided not to have your child get a shot for reasons other than illness or allergy?**
   1. Yes
   2. No
   3. I do not know
3. **If you had another infant today, would you want him/her to get all the recommended shots?**
   1. Yes
   2. No
   3. I do not know
4. **How sure are you that following the recommended shot schedule is a good idea for your child?**

Not at all sure 0 1 2 3 4 5 6 7 8 9 10 completely sure

1. **Children get more shots than are good for them**
   1. Strongly agree
   2. Agree
   3. Not sure
   4. Disagree
   5. Strongly disagree
2. **I believe that many of the illnesses shots prevent are severe / I believe that vaccines prevent severe illnesses**
   1. Strongly agree
   2. Agree
   3. Not sure
   4. Disagree
   5. Strongly disagree
3. **It is better for my child to develop immunity by getting sick than to get a shot.**
   1. Strongly agree
   2. Agree
   3. Not sure
   4. Disagree
   5. Strongly disagree
4. **It is better for children to get fewer vaccines at the same time.**
   1. Strongly agree
   2. Agree
   3. Not sure
   4. Disagree
   5. Strongly disagree
5. **How concerned are you that your child might have a serious side effect from a shot?**
   1. Very concerned
   2. Somewhat concerned
   3. Not sure
   4. Not too concerned
   5. Not at all concerned
6. **How concerned are you that any one of the childhood shots might not be safe?**
7. Very concerned
8. Somewhat concerned
9. Not sure
10. Not too concerned
11. Not at all concerned
12. **How concerned are you that a shot might not prevent the disease?**
13. Very concerned
14. Somewhat concerned
15. Not sure
16. Not too concerned
17. Not at all concerned
18. **Overall, how hesitant about childhood shots would you consider yourself to be?**
19. Very hesitant
20. Hesitant
21. Not sure
22. Not hesitant
23. Not hesitant at all
24. **I trust the information I receive about shots.**
    1. Strongly agree
    2. Agree
    3. Not sure
    4. Disagree
    5. Strongly disagree
25. **I am able to openly discuss my concerns about shots with my child’s doctor.**
    1. Strongly agree
    2. Agree
    3. Not sure
    4. Disagree
    5. Strongly disagree
26. **All things considered, how much do you trust your child’s doctor?**

Do not trust at all 0 1 2 3 4 5 6 7 8 9 10 completely trust

***Thanks a lot for your time***
